# The role of water intake in the severity of pain and menstrual distress among females suffering from primary dysmenorrhea: a semi-experimental study

**DOI:** 10.1186/s12905-021-01184-w

**Published:** 2021-01-28

**Authors:** Behnaz Torkan, Mahsasadat Mousavi, Samira Dehghani, Leila Hajipour, Narges Sadeghi, Marzieh Ziaei Rad, Ali Montazeri

**Affiliations:** 1 Community Health Research Center, Isfahan (Khorasgan) Branch, Islamic Azad University, Isfahan, Iran; 2 Department of Midwifery, School of Medical Sciences, Tonekabon Branch, Islamic Azad University, Tonekabon, Iran; 3 Population Health Research Group, Health Metrics Research Center, Iranian Institute for Health Sciences Research, ACECR, Tehran, Iran; 4 Faculty of Humanity Sciences, University of Science and Culture, Tehran, Iran

**Keywords:** Primary dysmenorrhea, Pelvic pain, Arginine vasopressin, Water intake, Dehydration

## Abstract

**Background:**

Dysmenorrhea is the most common health problem among women of reproductive age. The aim of the present study was to investigate the modifying role of water intake in menstrual distress and severity of pain among young female adolescents.

**Method:**

A semi-experimental study was conducted on a sample of undergraduate female students aged 18–30 years in Isfahan, Iran from 2016 to 2019. Volunteers who had history of suffering from primary dysmenorrhea and drank less than 1600 ml water per day were assigned into water intake (n = 70) and control (n = 70) groups. Participants could select the group in which they desired to be considered. The water intake group was asked to drink water regularly based on a protocol for two menstrual periods while the control group did not receive any form of intervention. Demographic information and menstrual characteristics and severity of menstrual pain (based on a visual analogue scale), were obtained using a short questionnaire. The data were compared between and within two groups before and after intervention using chi-square test, Mann–Whitney U test, and the Friedman’s analysis of variance.

**Results:**

The mean age (SD) of participants was 22.0 (2.7) years and 77 students reported normal duration of menstrual bleeding. The number of students who had normal duration of menstrual bleeding (4–6 days) in water intake group increased after intervention (39 vs. 49 after first and 46 after second cycles of menstruation). However, the interval of menstrual cycle did not change significantly in either groups. Considerable decrease in using pain killer was observed in water intake group (*p* < 0.001). No significant differences were observed between control and water intake groups before intervention in pain intensity (pain mean score 7.64 vs. 7.06), but within group comparison showed that pain intensity was significantly decreased among water intake group (*p* < 0.0001) while for control group only a significant decrease was observed for the first day of menstrual bleeding.

**Conclusion:**

The findings suggest that water intake might have modifying role in reducing menstrual bleeding duration, pain killer utilization, and pain intensity during menstrual period.

**Trial registration:**

IRCT20180708040377N1, 16 April 2020, Retrospectively registered, at https://www.irct.ir/trial/32446

## Background

Dysmenorrhea is the most common health problem in the reproductive years of women, and it is the first reason they seek gynecological care [1]. Dysmenorrhea is not a life-threatening disorder, but it affects women's performance in people who desire to have high and regular activities [2, 3]. The incidence of dysmenorrhea is reported between 16 and 93% [4]. A study conducted in Iran showed that about 70% of young women suffer from dysmenorrhea [5]. The symptoms of dysmenorrhea such as muscle cramp, headache, fatigue, nausea and vomiting, diarrhea and shivering can reduce the quality of life and social activities of women [1, 6].

Dysmenorrhea is classified into two groups of primary and secondary dysmenorrhea. Primary dysmenorrhea is defined as a painful menstruation occurring just before or during menstruation in people who have normal ovulation and normal pelvic organs [2]. Primary dysmenorrhea presenting with cyclic pain starts within 48 h of the first day of the menstrual cycle, and resolves by menstrual cycle day 2 or 3 [1]. Secondary dysmenorrhea is more common in 40 to 50 aged women and occurs in women with pathologic diagnosis [2].

Uterine activity in women with dysmenorrhea has an abnormal pattern. During menstruation, uterine endometrium produces and releases a large amount of prostaglandin F2a, etiological factor in primary dysmenorrhea, leading to uterine contraction, pain and cramps, hypoxia and ischemia of the uterus [7]. Compared with prostaglandin F2a, Other most important vasoconstrictor agents in the nonpregnant uterus seem to be endothelin-1 and noradrenaline and arginine vasopressin (AVP) [8]. Studies have shown the important role of AVP in the onset of uterine myometrium hyperactivity and decrease in uterine blood flow, leading to appearing symptoms of early dysmenorrhea [8, 9]. AVP, a potent myometrial contractile agent in vitro and in vivo, seems to exert its effect on the human uterus via AVP V1a and oxytocin receptors. The effect of AVP on the smooth muscle of arteries in non-pregnant uterus may also be pronounced [9].

A slight deficit of water very quickly activates AVP. In low plasma concentrations, AVP induces nearly maximal renal water conservation well before activation of thirst [8, 10]. Data indicates that there is no difference between the plasma osmolality of people who chronically drink either low or high amounts of water, but greater vasopressin is seen in low-drinkers [11]. Some animal studies have shown that creating dehydration in body by injection of hypertonic solution leads to the release of vasopressin [12]. A study conducted on the impact of infusion of hypertonic saline solution in women with dysmenorrhea, showed a slight increase in hormone, leading to an increase in uterine contractions and pain [8]. The results of a research study showed that drinking water during menstruation was associated with pain reduction and discomfort [13].

The prevalence of dehydration among adult people ranges from 16% to 28% [14] and water has been considered the most popular drinking among people over the age of 6 years. The average consumption of liquids including water in Iran is approximately 1.7 and 1.9 L per day in men and women, respectively [15]. The estimated total water intake in adults aged 20–54 years is 1307 mL/day. This amount is 1198 mL/day in senior adults in France, and 3563 mL/day in the USA [16]. Human water requirements should not be based on a ‘minimal’ intake, as this might eventually lead to a deficit and possible adverse performance and health consequences. Based on a large number of studies, daily consumption of at least 8 glasses of water can lead to the elimination of toxins and promote health [17].

However, the modifying role of water intake on dysmenorrhea and menstrual characteristics is not well documented. The present study aimed to investigate the role of water intake in menstrual distress and the severity of pain among females suffering primary dysmenorrhea.

## Methods

### Study design

A non-randomized semi-experimental controlled trial was conducted in Isfahan, Iran from March 2016 to March 2019. Iran National Committee for Ethics in Biomedical Research approved the study (IR.MUI.REC.1395.4). Since water drinking rate varies according to temperament, age, gender, body mass index, season, occupation and country [18, 19], this study was conducted among single female university students aged 18–30 years with normal body mass index (18.5–25 kg/m^2^) . In order to eliminate the effect of temperature on the amount of water consumed, sampling was done during spring and autumn.

### Participants

**Fig. 1:**
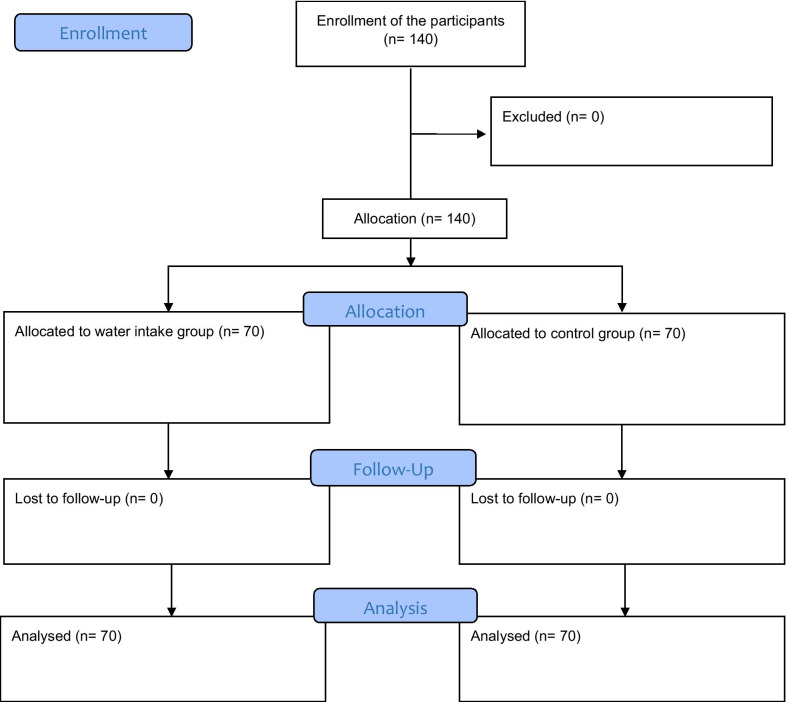
The study flowchart

The inclusion criteria were self-reporting of pelvic pain during the first 3 days of menstruation in 3 consecutive menstrual cycle over the last 6 months [19], daily intake of water less than1600 ml/day and having no symptoms of vaginal discharge during the study. Individuals were asked to record the number of glasses of water they drank daily for a period of 7–10 days. Those who proved to drink less than 1600 ml/day were selected for the study. The students were excluded if they were professional athletes or if they had chronic disorders such as diabetes, cardiovascular disorders, hepatic or renal disorders, infectious disease or epilepsy, and past diagnosis of having secondary dysmenorrhea or pelvic pathology as assessed by a health professional [2, 18]. Students who did not desire to continue the intervention, or who followed less than 80 percent of the protocol of drinking water (1600 ml/day) also were excluded. Written informed consent was obtained from the respondents. Convenience sampling was done and participants were assigned to intervention (water intake) and control groups based on their intention. Sampling continued until the intended number of participants for each group was achieved (Fig. 1).

### Sample size

The sample size was estimated based on pain reduction after intervention. The following formula was used to calculate the sample size [20]

$${\text{n}} = \left( {{\text{Z}}_{{\upalpha /2}} + {\text{Z}}_{\upbeta } } \right)^{2} *{\text{P }}\left( {1 - {\text{P}}} \right)*\left( {{\text{r}} + 1} \right)/\left( {{\text{P}}1 - {\text{P}}2} \right)^{2}$$

where the following parameters were considered: Z_α/2_ = 1.96 and Z_β_ = 0.842 (for 5% significance level and power 80%); P1 = 10% (pain reduction in control group), P2 = 30% (pain reduction in water intake group); r = 1 (equal cases per each group), P = P1 + rP2/r + 1 = 20%. As such we estimated that the study would require at least 63 female student per each group. Considering about 10% drop out a sample of 70 students per each group was thought.

### Intervention

Participants in the intervention group were asked to follow a proposed protocol of drinking water per day over the two menstruation cycles. Based on this protocol, they were asked to drink a glass of water containing 250 ml 30 min before breakfast, two glasses between breakfast and lunch intermittently, one glass 30 min before lunch, two glasses between lunch and dinner intermittently, one glass 30 min after dinner, and one glass 30 min before going to bed, giving a total of 2000 ml of water intake per day [21]. The participants in intervention group were asked to have a bottle containing 500 ml of water by themselves to be able to count the amount of water drank per day. The Control group were advised to continue drinking water based on their regular habit, and report the menstruation characteristics by completing the study questionnaire. To provide support and to prevent dropout, both groups were followed-up through social networks.

### Outcomes

The primary outcomes were recording of duration of menstrual bleeding, interval of mensuration cycle and the number of pain killer taken during menstruation. The secondary outcome was to assess pain intensity during the first 3 days of menstruation during three consequent menstrual cycles.

### Measures

In addition to demographic information both water intake and control groups were asked to record the following information in a self-designed questionnaire.

1. Duration of menstrual bleeding in days. The duration then was categorized into normal (4–6 days) and abnormal (≥ 7 days) menstruation [22].
2. Interval of menstruation cycles in days. The interval of menstruation was classified as short: ≤ 21 days, normal: 22–35 days, and long: ≥ 36 days [22].
3. The number of pain killer taken during menstruation. This was recorded as frequency of any pharmacological pain killer taken in order to relieve pelvic pain and categorized as 0, 1–2, and 3–4.
4. The intensity of menstrual pain: This was measured using a Visual Analog Scale (VAS). The VAS is commonly used to measure menstrual pain intensity and consists of a 10-cm horizontal line with ‘no pain’ on one end and ‘worse possible pain’ on the other end [6]. Self-reported pain is the single most important element in understanding pain [23].

All participants completed the questionnaire before intervention, and at the first 3 days of menstruation during three consequent menstrual cycles (before intervention, the first and the second menstrual cycle after intervention) as applied.

### Statistical analysis

The data extracted from the questionnaires were statistically analyzed using the SPSS 22. Descriptive statistics including frequency, mean and standard deviation were used to describe the data. In addition to Chi-square, t-test was used for comparison of quantitative demographic characteristics. Also duration and interval of menstruations; and the number of pain reliever taken were compared between two groups using Chi-square test. Since for some values normality was not confirmed (Kolmogorov–Smirnov test), Mann–Whitney U-test was applied to compare intensity of menstrual pain between two groups. In addition, within group comparison was performed using related samples Friedman’s two-ways analysis of variance. In all instances the level of statistical significance was set at *p* < 0.05%.

## Results

### Characteristics of participants

In all, 140 female students were entered into the study (water intake = 70 and control group = 70). No significant differences were observed between two groups for the matched characteristics such as age, age at menarche, initiation of dysmenorrhea and menstrual regulation. The characteristics of the study samples are presented in Table 1.

**Table 1:** The demographic and menstrual characteristic of female students who suffered from primary dysmenorrhea in the water intake and control groups

	Control group (n = 70)	Water intake group (n = 70)	*p* value
Age (mean, SD)	21.6 (2.31)	22.4 (3.02)	0.10*
Menarche age (mean, SD)	12.8 (1.08)	13.2 (1.65)	0.10*
Age of menstrual pain initiation (mean, SD)	14.4 (2.28)	15.1 (2.29)	0.09*
Body Mass Index (mean, SD)	21.1 (2.47)	20.7 (2.04)	0.24*
*Menstrual regularity (number, %)*			0.18**
Regular	55 (78.6)	61 (87.1)	
Irregular	15 (21.4)	9 (12.9)	
*Marital status (number, %)*			0.66**
Single	56 (80)	58 (82.9)	
Married	14 (20)	12 (17.1)	
*Faculty (number, %)*			0.21**
Medical sciences	50 (71.4)	43 (61.4)	
Non-medical sciences	20 (28.6)	27 (38.6)	

*t-test

**Chi-square test

### Duration of menstrual bleeding

No significant difference was observed between control and water intake groups in duration of menstrual bleeding before and after the first cycle of intervention (*p* = 0.094). However, the menstrual bleeding duration in intervention group significantly decreased as compared to the control group in the second cycles of menstruation (*p* = 0.043). The results are presented in Table 2.

**Table 2:** Duration of menstrual bleeding before and after intervention in water intake and control groups

	Duration of menstrual bleeding*	Control group (n = 70)	Water intake group (n = 70)	*p* value**
		Number (%)	Number (%)	
Before intervention				0.125
	Normal	38 (54.3)	39 (55.7)	
	Abnormal	32 (45.7)	31 (44.3)	
The first menstrual cycle after intervention				0.094
	Normal	41 (58.6)	49 (70.0)	
	Abnormal	29 (41.4)	21 (30.0)	
The second menstrual cycle after intervention				0.043
	Normal	37 (52.9)	46 (65.7)	
	Abnormal	33 (47.1)	24 (34.3)	

*Normal: 4–6 days, Abnormal: ≥ 7 days

**Chi-square test

### Menstruation interval

There were no significant differences between study groups before and after intervention in terms of menstruation intervals. However, number of participant in water intake group who reported normal interval of menstruation cycle increased after intervention. The control group did not experience any significant changes in menstrual cycle length at three points assessments. The results are shown in Table 3.

**Table 3:** Menstrual bleeding interval before and after intervention in water intake and control groups

	Length of menstruation cycle*	Control group (n = 70)	Water intake group (n = 70)	*p* value**
		Number (%)	Number (%)	
Before intervention				0.112
	Short	16 (22.9)	22 (31.4)	
	Normal	53 (75.7)	45 (64.3)	
	Long	1 (1.4)	3 (4.3)	
The first menstrual cycle after intervention				0.321
	Short	15 (21.4)	18 (25.7)	
	Normal	55 (78.6)	52 (74.3)	
	Long	0 (0.0)	0 (0.0)	
The second menstrual cycle after intervention				0.149
	Short	16 (22.9)	22 (31.4)	
	Normal	54 (77.1)	45 (64.3)	
	Long	0 (0.0)	3 (4.3)	

*Short: ≤ 21 days, Normal: 22–35 days, Long: ≥ 36 days

**Chi-square test

### Pain killer

There were no significant differences between control and water intake groups in number of pain relievers used before intervention (*p* = 0.066), but a significant difference was observed during the first and the second cycles of menstruation after intervention (*p* < 0.001). The results are shown in Table 4.

**Table 4:** Frequency of taking pain reliever before and after intervention in water intake and control groups

	Number of pain reliever	Control group (n = 70)	Water intake group (n = 70)	*p* value*
		Number (%)	Number (%)	
Before intervention				0.066
	0	4 (5.7)	7 (10.0)	
	1–2	53 (75.7)	51 (72.9)	
	3–4	13 (18.6)	12 (17.1)	
The first menstrual cycle after intervention				< 0.001
	0	5 (7.1)	5 (7.1)	
	1–2	51 (72.9)	65 (92.9)	
	3–4	14 (20.0)	0 (0.0)	
The second menstrual cycle after intervention				< 0.001
	0	4 (5.7)	28 (40.0)	
	1–2	55 (78.6)	42 (50.0)	
	3–4	11 (15.7)	0 (0.0)	

*Chi-square test

### Pain intensity

No significant differences were observed between control and water intake groups before intervention in terms of the intensity of menstruation pain during the first 3 days of menstruation (*p* > 0.05). However, the water intake group reported significantly less menstrual pain during the first 3 days of menstruation both in the first and the second menstrual cycle after intervention. Finally, within group comparison showed that pain intensity was significantly decreased among water intake group while for control group only a significant decrease was observed for the first day of menstrual bleeding. The results are presented in Table 5.

**Table 5:** Comparison of menstrual pain intensity in two groups before, at the first and at the second stage of the intervention as measured by the visual analog scale

Intensity of menstrual pain	Stage	Control group (n = 70)	Water intake group (n = 70)	*p* value*
		Mean (SD)	Mean (SD)	
The first day of menstrual bleeding				
	Before intervention	7.64 (2.16)	7.06 (2.21)	0.06
	The first menstrual cycle after intervention	7.36 (1.87)	5.84 (2.22)	0.007
	The second menstrual cycle after intervention	7.67 (1.92)	5.06 (2.43)	< 0.001
	*p* value**	0.004	< 0.0001	
The second day of menstrual bleeding				
	Before intervention	4.94 (2.19)	4.43 (2.86)	0.176
	The first menstrual cycle after intervention	4.57 (1.95)	3.50 (2.43)	0.058
	The second menstrual cycle after intervention	4.71 (2.04)	3.34 (2.97)	0.385
	*p* value**	0.148	< 0.0001	
The third day of menstrual bleeding				
	Before intervention	2.20 (1.58)	2.61 (2.87)	1.000
	The first menstrual cycle after intervention	2.13 (1.38)	1.67 (2.34)	0.486
	The second menstrual cycle after intervention	2.43 (2.42)	1.29 (1.61)	0.004
	*p* value**	0.485	< 0.0001	

*Mann–Whitney U t-test

**Related samples Friedman’s two-ways analysis of variance

## Discussion

The results of this semi-experimental trial suggest that drinking 1600–2000 ml of water daily and regularly can alleviate the severity of primary dysmenorrhea, shorten the length of menstrual bleeding and reduces the average number of pharmacological pain relievers took during menstruation. Our findings concur with a previous study that revealed improvements in severity of menstrual pain in women who had water load at 90th minute after starting of dysmenorrhea [13].

According to pain theory, arrangement of pain is cyclic and includes the following order: pain/tension/fear/pain [24]. Since drinking water plays a significant role in reduction of vasopressin concentration, it seems that taking more water can be effective in reducing uterine contraction, diminishing tension and fear, and can be considered as a natural painkiller in the body.

We found that recommended amount of water intake decreased the pelvic pain of dysmenorrhea after intervention. Furthermore, a significant improvement in menstrual pain was observed in the second cycle of intervention compared with the first cycle of intervention. This improvement could be the result of better adjustment to water intake protocol over the second cycle of intervention. Data indicated that in the first cycle of intervention, most of the participants, obeyed 80% of the protocol (consumed about 1600 ml of water per day) while the majority of the participants reached their daily water intake to 1800-2000 ml/d over the second cycle of intervention. Results also showed that intervened group consumed less pain relievers during dysmenorrhea after intervention, but their length of menstrual bleeding did not change. Participants did not encounter any specific side-effects during intervention. Two of the participants claimed positive effects of drinking water including less acne and brighter appearance. The effect of modifying water intake on the menstruation bleeding did not investigated since it needed to be considered as a single study.

The participants did not receive any follow-up assessments after intervention. Further studies with double-blinded trial should be considered to investigate the effect of continual water intake modification on the variables in larger sample size. Future studies should also compare modifying water intake with other interventions, such as aerobic exercise and lifestyle modifications (eating behavior, regular physical activity, self-care, high-level of social relationships, and reduction of stress levels) to compare their benefits. There is evidence that risk factors such as drinking cola drinks, duration of the menstrual flow, eating meat and having a first-degree relative could affect dysmenorrhea [25].

This study is the first semi-experimental trial that investigated the modification role of water intake on severity of pelvic pain and menstrual distress in students with primary dysmenorrhea. We believe the findings could contribute to existing knowledge on the topic and perhaps help young women to overcome the pelvic pain of dysmenorrhea by modifying the pattern of drinking daily water. However, the data for this study was self-reported and obtained from females studying at Isfahan (Khorasgan) Azad University in Iran, which therefore restricts the extrapolation of the results to the wider populations. Thus the authors recommend other studies to be conducted in more diverse populations. In addition, one should note that we collected limited information for the study while there might be other confounding variables such as diet and alcohol consumption that potentially could influence primary dysmenorrhea [26, 27].

## Conclusion

Overall, the findings suggest that water intake could decrease the duration of menstrual bleeding, the amount of pain relievers consumed, and the severity of pelvic pain among the young women suffering from primary dysmenorrhea and drinking less than 1600mml of water per day.

## Data Availability

The dataset used and analyzed during the current study are available from the corresponding authors upon making official request.

## Abbreviations

AVP: Arginine vasopressin
VAS: Visual Analog Scale
SD: Standard deviation
